# Care pathways in older patients seen in a multidisciplinary same day emergency care (SDEC) unit

**DOI:** 10.1093/ageing/afad257

**Published:** 2024-01-25

**Authors:** Tania C N Elias, Chloe Jacklin, Jordan Bowen, Daniel S Lasserson, Sarah T Pendlebury

**Affiliations:** Departments of Acute Internal Medicine and Older Persons' Services, Great Western Hospital NHS Foundation Trust, Swindon SN3 6BB, UK; Departments of Care of the Elderly and Stroke Medicine, North Middlesex University Hospital NHS Trust, Sterling Way, London N18 1QX, UK; Department of Acute Internal Medicine and Geratology, Oxford University Hospitals NHS Foundation Trust, John Radcliffe Hospital, Oxford OX3 9DU, UK; Department of Acute Internal Medicine and Geratology, Oxford University Hospitals NHS Foundation Trust, John Radcliffe Hospital, Oxford OX3 9DU, UK; NIHR Applied Research Collaboration (ARC) West Midlands, Warwick Medical School, University of Warwick, Coventry, Warwickshire CV4 3AL, UK; Department of Acute Medicine, City Hospital, Sandwell and West Birmingham Hospitals NHS Trust, Birmingham B18 7QH, UK; Department of Acute Internal Medicine and Geratology, Oxford University Hospitals NHS Foundation Trust, John Radcliffe Hospital, Oxford OX3 9DU, UK; NIHR Oxford Biomedical Research Centre, John Radcliffe Hospital, Oxford University Hospitals NHS Foundation Trust, Oxford OX3 9DU, UK; Nuffield Department of Clinical Neurosciences, Wolfson Centre for Prevention of Stroke and Dementia, John Radcliffe Hospital, and the University of Oxford, Oxford, UK

**Keywords:** ambulatory care, same day emergency care (SDEC), frailty, acute care, outcomes, follow-up, older people

## Abstract

**Background:**

Same day emergency care (SDEC) services are being advocated in the UK for frail, older patients in whom hospitalisation may be associated with harm but there are few data on the ‘ambulatory pathway’. We therefore determined the patient pathways pre- and post-first assessment in a SDEC unit focussed on older people.

**Methods:**

In consecutive patients, we prospectively recorded follow-up SDEC service reviews (face-to-face, telephone, Hospital-at-Home domiciliary visits), outpatient referrals (e.g. to specialist clinics, imaging, and community/voluntary/social services), and hospital admissions <30 days. In the first 67 patients, we also recorded healthcare interactions (except GP attendances) in the 180 days pre- and post-first assessment.

**Results:**

Among 533 patients (mean/SD age = 75.0/17.5 years, 246, 46% deemed frail) assessed in an SDEC unit, 210 were admitted within 30 days (152 immediately). In the 381(71%) remaining initially ambulatory, there were 587 SDEC follow-up reviews and 747 other outpatient referrals (mean = 3.5 per patient) with only 34 (9%) patients being discharged with no further follow-up. In the subset (n = 67), the number of ‘healthcare days’ was greater in the 180 days post- versus pre-SDEC assessment (mean/SD = 26/27 versus 13/22 days, *P* = 0.003) even after excluding hospital admission days, with greater healthcare days in frail versus non-frail patients.

**Discussion and Conclusion:**

SDEC assessment in older, frail patients was associated with a 2-fold increase in frequency of healthcare interactions with complex care pathways involving multiple services. Our findings have implications for the development of admission-avoidance models including cost-effectiveness and optimal delivery of the multi-dimensional aspects of acute geriatric care in the ambulatory setting.

## Key Points

- Pathways after initial same day emergency care (SDEC) assessment were complex and highly variable with frequent escalations and de-escalations in care (outpatient to inpatient, community hospital to acute hospital, and vice-versa).
- Among patients remaining ambulatory after index assessment, only 9% were discharged immediately with no further follow-up and the remaining patients had on average 3.5 reviews/appointments each.
- SDEC assessment was associated with a 2-fold increase in the number of days involving any sort of healthcare interaction in the 180 days after versus before assessment.
- The number of days with a healthcare interaction was greater in frail versus non-frail patients both before, and after, SDEC assessment.

## Introduction

Older adults with frailty comprise an increasing proportion of patients referred to hospital with acute medical problems, and may have conditions that could be treated without admission [1, 2] potentially avoiding harm associated with hospitalisation [3, 4]. The last decade has therefore seen an increasing UK focus on provision of care which minimises in-patient hospital stays [5–7]. This trend has gained further impetus amidst increasing pressures on healthcare systems and the Covid-19 pandemic.

UK policy has encouraged an ‘ambulatory by default’ approach with development of service models integrating comprehensive geriatric assessment (CGA) and treatment for those with frailty to reduce avoidable hospital admission and facilitate discharge [8]. Services may be based in hospital (e.g. Same Day Emergency Care (SDEC), frailty units, frailty discharge teams within the Emergency Department-ED) or in community settings (e.g. Hospital-at-Home, Community Rapid Response), and where data exist, some report similar outcomes to hospital admission or reductions in admission rates [8–10].

However, little is known about frail patients assessed in SDEC services, and the evidence available reveals a nuanced picture. While SDEC may reduce hospital admissions in carefully-selected older patients [11, 12] we have previously shown that in a multidisciplinary SDEC unit managing conditions generally seen in acute medical services, there is a high incidence of (<30 days) hospital admission particularly in those with physical or cognitive frailty [13]. Furthermore, care pathways for individuals with frailty who avoid initial hospital admission following acute ambulatory care assessment have not been described. SDEC is often thought of as providing ‘same day care’ in which assessment and treatment is completed in one visit, possibly because of studies from SDEC services selecting by condition (e.g. pulmonary embolism) [14, 15]. Yet studies in Emergency Departments and acute medical units have demonstrated high resource use in older and frail patients discharged after assessment, with higher levels of readmission [16–20].

Better understanding of how SDEC for older patients with frailty interacts with hospital and community services, at the point of assessment and beyond, is essential for the effective design, planning, and resourcing of such services. We therefore undertook a prospective study within the setting of a specialist-led, multidisciplinary community hospital-based SDEC centre, the Abingdon Emergency Multidisciplinary Unit (EMU [13, 21]). Whilst this study pre-dated the development of widespread medically-led Acute Hospital-at-Home models and NHS England’s Virtual Ward programme, the detailed characterisation of the cohort and of ongoing needs/service use is generalizable to a range of services developed as an alternative to hospital admission. We aimed to (i) determine the number and type of SDEC reviews and planned other healthcare encounters following initial SDEC assessment and (ii) describe in depth the community and secondary healthcare use in a subset of this cohort for the 180 days before and 180 days after the EMU encounter.

## Methods

### Setting

The EMU is based in a community hospital located approximately 10 miles from the main regional acute hospital run by the Oxford University Health NHS Foundation Trust-OUHFT. The EMU was designed to address the needs of frail, older patients with acute illness. Patients are accepted by EMU clinicians after discussion with the referring GP or other healthcare professional. The EMU patients are therefore more similar to those seen in an acute medical take than the undifferentiated ED population with a broadly consistent spectrum of presenting complaints [13] but patients with high acuity, acute coronary syndromes or stroke are redirected to the acute hospital. EMU has six assessment cubicles and two rooms and is staffed 7 days a week. The multidisciplinary team includes an occupational therapist/physiotherapist, social worker/discharge coordinator, five to seven nurses/health care assistants, one trainee doctor, and is led by a geriatrician or senior interface physician (with hospitalist and community skillsets). Point-of-care blood testing [22] and plain X-ray are available. Other imaging is arranged at the acute hospitals. Intravenous treatments (including blood products) may be administered.

After assessment in EMU, patients may be:

- discharged without further review;
- discharged with planned further EMU-review (face to face, telephone or Hospital-at-Home team for intravenous treatments and phlebotomy);
- referred to other out-patient hospital- or community-based services;
- admitted to a community or acute hospital bed.

### Patient cohort

All consecutive patients assessed in EMU August–December 2015 were included. There were no exclusion criteria. Data on the methodology, case-mix and factors associated with hospital admission have been published previously [13, 21]. Patients were prospectively assessed by EMU clinicians (JB,TE) using a structured paper clerking proforma [23] including the question ‘Do you judge the patient to be frail?’. Functional status was defined by the Barthel index [24] and modified Rankin score [25]. Comorbidity was defined by the Charlson index [26].

The OUHFT Divisional Management approved this study (Datix Numbers-3812, 7147). Pseudonymised data were entered into the Oxford Cognitive Co-morbidity and Frailty Ageing Research Database (ORCHARD) which was set up to provide data for audit and research (research ethics committee reference 18/SC/0184 [27]).

### Outcomes

Ambulatory versus non-ambulatory status was defined as living at home vs any hospital admission within 30 days from EMU first assessment. An immediate admission was defined as an admission on the day of first assessment, and a delayed admission was defined as any other admission <30 days. Inter-hospital in-patient transfers were recorded. Non in-patient healthcare interactions related to the index problem were obtained prospectively via EMU attendance or from hospital and community electronic patient records (EPRs). Data were unavailable for eight patients who were resident out of area. Healthcare interactions were recorded as follows:

- Completed EMU service reviews (face-to-face review, telephone call, Hospital-at-Home visit)
- Referral to or appointment with: other specialist, hospital clinic, diagnostics (imaging, endoscopy, cardiac physiology), GP/district nursing, community-led clinics/services, community rehabilitation, social services/care agency, voluntary sector

For the subset of the first 67 patients assessed in the cohort, all hospital and community inpatient and outpatient healthcare interactions were recorded for 180 days pre- and post- first EMU assessment from searches of hospital and community EPRs. Healthcare interactions involving secondary care, community healthcare, allied healthcare professional and social care appointments (e.g. community clinics, podiatry, palliative care, district nursing, physiotherapy, occupational therapy, voluntary sector) were noted with dates; the number of days with at least one healthcare interaction in the 180 days before and the 180 days after was determined including days in hospital. GP appointment data were not available.

### Statistical analyses

Any patient re-referred with a new illness episode during the study was included as a new case. The clinicians’ judgement of frailty was used to define frail and non-frail groups. Comparison of the clinical characteristics of the first 67 patients and the entire cohort, and of follow-up appointment type in frail versus non frail patients were made using independent t-tests and chi-square test as appropriate. All data were analysed using IBM SPSS v28 software.

## Results

**Figure 1:**
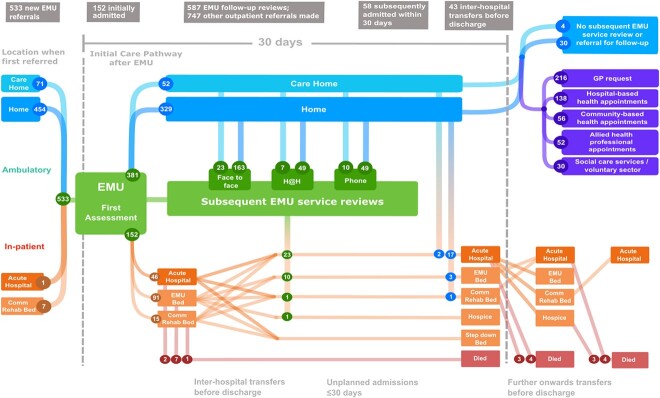
Clinical pathways of patients assessed in EMU showing the location of patients at the time of referral, and subsequent care pathway including hospital admission and inter-hospital admissions, EMU reviews and other planned healthcare interactions.

Five hundred and thirty-three consecutive patients were referred to EMU over the four-month study period, the vast majority (498, 93%) by their GP or paramedics (Table 1, Figure 1). The majority (454, 85%) were resident in their own home of whom 172 (38%) received either formal or informal care with the remainder being referred from care homes (71, 13%), or the regional acute hospital or a community hospital (8, 2%). Overall, 246 (46%) were deemed to be frail. The dedicated EMU transport service was used by 152 (29%) patients at least once. There was no difference between the subset of the first 67 patients and the entire cohort in age, gender, care needs, Charlson co-morbidity index, functional status, frailty, delirium or admission <30 days (Table 1).

**Table 1:** Characteristics of the EMU cohort and subset of the first 67 patients

	All *N = 533*	Subset *N = 67*	*P* value
Age, mean/SD years	75.0/17.5	77.0/15.2	0.31
Gender, female	315 (59)	36 (54)	0.34
Referred by GP	443 (83)	54 (81)	0.68
Referred by paramedic	55 (10)	10 (15)	0.21
Referred other route^*^	35 (7)	3 (4)	0.35
Resident in care home	71 (13)	7 (10)	0.49
Required EMU transport at least once	152 (29)	26 (39)	0.08
Charlson Comorbidity Index >3	395 (74)	52 (78)	0.48
Barthel Index <20	299 (56)	33 (49)	0.23
Premorbid modified Rankin Scale >2	193 (36)	39 (58)	0.50
Clinical impression of frailty	246 (46)	30 (45)	0.79
Delirium at first assessment	87 (16)	13 (19)	0.47
SIRS>1	159 (30)	18 (27)	0.62
NEWS>4^*^^*^	102 (19)	11 (16)	0.55
Remained ambulatory at 30 days	315 (60)	37 (55)	0.54
Receiving EMU follow-up (any type)	227 (43)	29 (43)	0.92
Immediate admission after first assessment	152 (29)	22 (33)	0.46
Delayed admission within 30 days	58 (11)	8 (12)	0.81

^*^There were 4 ‘walk-in’ patients who self-presented and were seen but this was not the standard route of access to the service.

^**^Denominator was 525. For 8 patients, 30-day outcome was unavailable. Numbers are n (%) unless otherwise specified. EMU = Emergency Medical Unit, SIRS = Systemic Inflammatory Response Syndrome. NEWS = National Early Warning Score.

**Figure 2:**
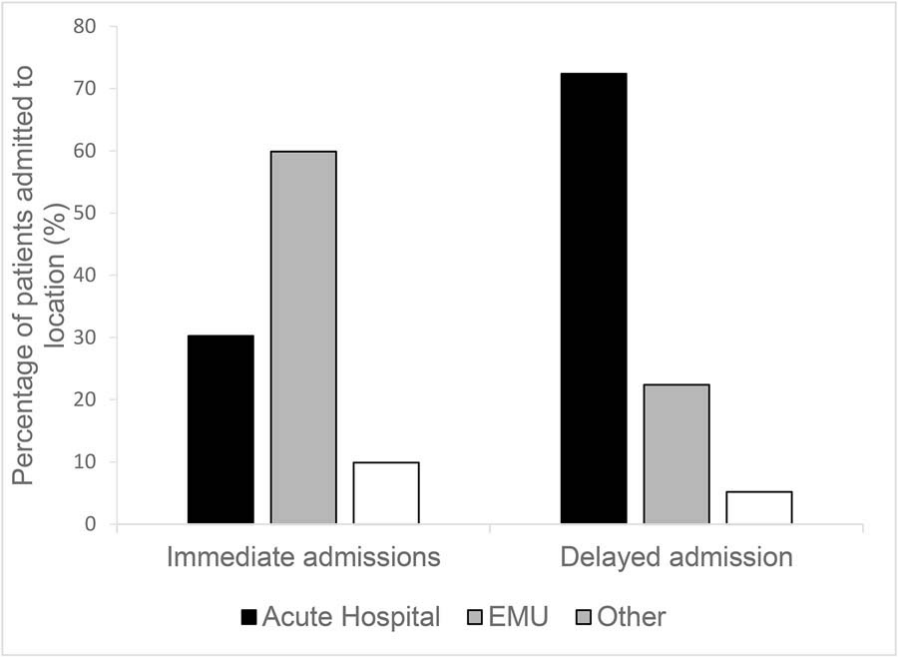
Comparison of admission location for immediate and non-immediate admissions following EMU assessment. Black bars = acute hospital admissions, grey bars = EMU admissions and white bars = admissions to other locations.

The care pathways followed by patients after initial EMU assessment were variable and highly complex (Figure 1). Among the 533 patients, 152 (29%) were admitted to hospital immediately; of these, 91 (59%) went to one of the EMU community hospital beds, 46 (30%) went to the regional acute hospital, and 15 (10%) went to another community hospital. A further 58 patients were subsequently admitted to hospital <30 days from the initial assessment. In contrast to those admitted immediately, these patients were more likely to be admitted to the regional acute hospital (42 (72%), *P* < 0.001) with only 13 admitted to an EMU bed, two to a community hospital and one to a hospice bed (Figure 2).

For the patients that were immediately admitted (n = 152), the predominant reasons (more than one for some patients) were: clinical need other than ‘confusion’ or delirium (n = 69), nursing need (n = 47), a mobility issue (n = 53), confusion/delirium (n = 23), carer anxiety (n = 10), pain control (n = 9) or being ‘unsafe’ to remain in the community (n = 15). For the 69 patients with a clinical need, admission to the acute hospital was more likely than other locations (41 versus 28), whereas for patients admitted for nursing need, mobility, confusion and/or delirium, or being ‘unsafe’ without clinical need (n = 83), admission to an EMU bed was more likely (65 to EMU versus 13 to other community hospitals and 5 to the acute hospital, *P* < 0.001). At least one inter-hospital transfer occurred in 33 (22%) of those initially admitted and seven (5%) had more than one transfer. Two of the 46 patients admitted to the acute hospital were subsequently stepped down to a community hospital, while of the 106 patients initially admitted to EMU or other community hospital, 12 were stepped up to an acute bed (and, of those, five were later transferred back to a community hospital), 17 moved to a different community hospital, two to a hospice, and two to a ‘step-down’ bed for nursing care. Inter-hospital transfers were also common in those with delayed admission (Figure 1).

Of the 381 (71%) patients who remained ambulatory after first assessment, only 34 (9%) patients were discharged with no further follow up either with EMU or any other service (Table 2). Overall, there were 587 EMU follow-up reviews and 747 other outpatient referrals (mean = 3.5 per patient). At least one further review by EMU services occurred in 227 (60%) of ambulatory patients (Table 2) and 67 had more than one type of review. The median (IQR) total EMU reviews (of any type) per patient was 2 (1–3), range 0–15; 107 (47%) had one, 48 (21%) had two, 23 (10%) had three, and 49 (22%) had ≥four (Appendix Figure 1). Planned interactions with other services included GP follow-up/action (216, 57%), hospital-based appointments (138, 36%), community-based health services (56, 15%), allied health professional appointments (52, 14%), social care services or voluntary sector agencies (29, 8%; Table 2). Frail patients were more likely to be admitted immediately (102 (41%) versus 50 (17%), *P* < 0.001) or within 30 days following first EMU assessment (38 (15%) versus 20 (7%), *P* < 0.001) but of those remaining ambulatory, the proportion with EMU follow-up was similarly high for those with and without frailty. However, frail patients were more likely to need EMU transport and to be referred to hospital at home services, community-based health appointments, allied health professionals and social/voluntary services (Table 2).

**Table 2:** Care pathways following EMU assessment for all and by clinical impression of frailty

Type of follow-up appointment arranged	All N = 533	Frail N = 246	Non-frail N = 287	*P* value
**Admitted after first assessment**	152	102 (41%)	50 (17%)	<0.001
**Ambulatory after first assessment** ^*^	381	144 (59%)	237 (83%)	<0.001
No follow-up	34	9 (6%)	25 (11%)	0.10
Any EMU follow-up	227	89 (62%)	138 (58%)	0.44
EMU face-to-face follow-up	186	75 (52%)	111 (47%)	0.34
EMU telephone follow-up	59	20 (14%)	39 (16%)	0.60
EMU Hospital-at-Home	56	28 (19%)	28 (12%)	0.05
GP specific follow-up	216	84 (58%)	132 (56%)	0.70
Hospital-based health appointment Outpatient clinics, elective procedures, hospital diagnostics e.g. radiology, cardiac physiology, endoscopy	138	44 (31%)	94 (40%)	0.08
Community-based health appointment District nurse, speciality nurse-led community services e.g. heart failure, diabetes, palliative care, podiatry, continence, falls	56	31 (22%)	25 (9%)	<0.001
Allied healthcare professional appointment Physiotherapy, occupational therapy, speech and language therapy, dietetics	52	30 (21%)	22 (8%)	<0.001
Social care services or voluntary sector agencies^*^^*^ Reablement services, new packages of care etc	29	18 (13%)	11 (5%)	0.005
**Admitted within 30 days**	210	140 (57%)	70 (24%)	0.001
**Ambulatory at 30 days**	315	104 (42%)	211 (74%)	<0.001
**Needing EMU transport at least once**	152	91 (37%)	61 (21%)	<0.001

^*^In all analyses in plain type below, the denominator is those remaining ambulatory after first assessment.

^**^Nine patients were referred to voluntary services for befriending or shopping assistance. EMU = Emergency Medical Unit. GP = General Practitioner

**Figure 3:**
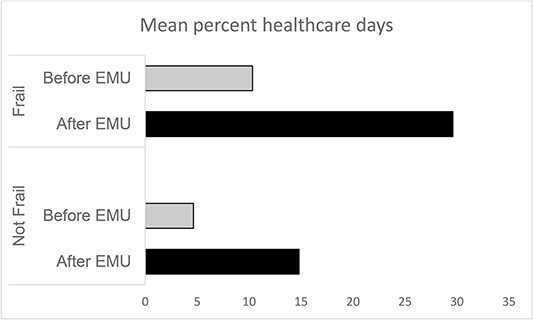
Mean percentage of ‘healthcare days’ in the 180 days before and after EMU assessment, in frail and not frail patients. The percentage of healthcare days was calculated by determining the number of days with at least one healthcare interaction of any type divided by the 180 day period censored at the time of death where relevant for the post-EMU period.

In the subset of the first 67 patients, 37 remained ambulatory, 22 were immediately admitted to hospital and eight had a non-immediate admission within 30 days. The mean number of healthcare interactions in the 30 days from the EMU assessment was 4.1 in those who remained ambulatory (compared to the 3.5 planned healthcare interactions plus EMU reviews for the whole cohort). The mean/SD number of ‘healthcare days’ was greater in the 180 days post- versus pre-EMU first assessment (26/27 versus 13/22 days, *P* = 0.003) even after excluding hospital admission days (mean/SD = 14/18 versus 5/7, *P* < 0.001). Frail patients (n = 30) had a greater number of healthcare days than non-frail patients (n = 37) both in the 180 days before (mean/SD = 19/25 versus 8/18 days, *P* = 0.04) and after EMU first assessment (mean/SD = 34/32 versus 20/21 days, *P* = 0.04). This meant that frail, compared to non-frail patients, had double the percentage of living days that were ‘healthcare days’ both before (mean/SD = 10/14% versus 5/10%) and after (30/28% versus 15/20%) EMU assessment (Figure 3).

## Discussion

In this cohort study from a multidisciplinary SDEC unit focussed on older people with high levels of frailty, with a patient population broadly similar to that seen in an acute medical unit, we examined the patient pathways following initial SDEC assessment on which there are few existing data. We found highly complex and variable pathways and frequent escalations and de-escalations in levels of care (outpatient to inpatient, community hospital to acute hospital, and vice-versa) suggesting a high level of need and associated patient burden.

The SDEC unit population was broadly similar to that of the regional acute medical unit [13, 23]. Overall, the mean age of the EMU patients was slightly older (75 years) than the regional acute medicine unit patients (72 years) but younger than the regional acute medicine population aged ≥65 years (82 years). Rates of care home residence (13%) were similar to that reported in acute medicine admissions (around 11% in patients aged >65–70 years [23, 28]). Rates of acuity as evidenced by NEWS/SIRS (20–30%) and delirium (16%) were slightly lower than in acute medical admissions [23] but standard acuity measures may not reflect severity of illness in older frail patients.

For those avoiding hospital admission after first assessment, less than a tenth had no referral for any type of follow-up. Nearly two-thirds underwent at least one subsequent review by the SDEC unit, much higher than reported from an SDEC unit in an acute hospital setting with mean patient age of <60 years [29] where only around one quarter had an SDEC unit follow-up appointment, despite similar listed presenting problems. The detailed subset data showed that on average, patients had four further healthcare interactions of any type within the 30 days after EMU assessment and this was sustained throughout the 180 days after initial assessment: one-fifth of days involved a healthcare interaction with greater burden in frail patients. Notably, the number of days with a healthcare interaction was 2-fold greater in the 180 days after versus before SDEC assessment for all patients, regardless of frailty status or whether or not they were admitted. Although this increase may reflect continued healthcare requirements related to the index problem or related declining health, this could also result from recognition of needs following CGA as shown previously in other settings [17, 18].

The patient pathway was no less complex in those admitted to hospital. Over one quarter in our study overall and two fifths of those with frailty were admitted immediately, which is lower than in an Emergency Department study of CGA (61% admitted,≥85 years [30]) but higher than in an acute hospital SDEC unit managing similar acute medical conditions to EMU in a younger population (14% [29]). A further 15% of those who were initially treated on an ambulatory pathway from EMU were admitted <30 days, which is similar to the readmission rate after hospital discharge for adults in England over a similar time period (14.4%) [31]. However, immediate admission was more often to an EMU bed whereas delayed admission was more often to the acute hospital possibly because of clinical acuity, onset of a new problem, or referral/self-referral directly to an Emergency Department. Transfers between different hospitals were frequent with implications for patient experience and continuity of care particularly in those with delirium/cognitive impairment.

Our findings show that for the vast majority of largely older and often frail patients, SDEC in a multidisciplinary unit with a patient population approximating that of the medical take is not a ‘one stop shop’. The prominence of healthcare interactions, along with dependence on other services, such as transport, is consistent with studies on frailty in other settings [32–36] and has implications both for individuals/families as well as for service development and resourcing. A previous ethnographic study on individual experiences of care in EMU, revealed that while views were generally positive, some found the long days and repeated attendances tiring [37]. In addition, onward referral across a myriad of different secondary care and community services as well as repeated SDEC unit visits may also lead to fragmentation of care and loss of oversight from a single senior decision maker with potential implications for quality, safety, and costs.

Our study has several strengths. We have filled an existing evidence gap on the nature and frequency of healthcare utilisation following assessment within a multi-disciplinary SDEC unit designed around the needs of older patients with conditions broadly similar to those seen in acute medical services [13, 21, 23]. We collected detailed prospective clinical data on a consecutive cohort including mapping of patient pathways before and after SDEC assessment in a subset. Such data are not available in administrative ‘Big Data’. Prospective data collection was led by a clinician (TE) embedded in EMU and enhanced by hand-searching of medical records likely more reliable than self-report or Medicare claims [38].

Limitations include the single centre setting, although the case-mix was broadly representative of emergency acute internal/geriatric medicine (in contrast to highly selective SDEC units [11, 12, 14, 15]). Second, availability of support services facilitating admission avoidance may differ in other regions. Third, quantitative frailty scoring was not done but the clinical impression of frailty was assigned by geriatrics-trained clinicians and was consistent with the presence of frailty markers [13]. Fourth, the subset, while containing rich data, was small, and did not include some appointments owing to different data systems resulting in under-estimation of healthcare interactions. In addition, we were unable to determine whether the high rates of follow-up were because of needs related to the index condition or to previously unidentified unmet needs. Fifth, data collection occurred prior to wider UK introduction of Hospital-at-Home services/virtual wards. However, the EMU service was progressive at inception in having a linked Hospital-at-Home service and in-house follow-up able to undertake some hospital level processes of care and the service remains broadly unchanged, confirming its ongoing relevance in the face of rising system pressures. Furthermore, the EMU experience directly informed current policy on SDEC and virtual wards although provision of Hospital-at-Home and virtual wards remains patchy nationally [39].

## Conclusion

Older patients with acute illness assessed in a multidisciplinary SDEC unit experience complex care pathways with diverse health/social care interactions even when hospital admission is avoided. Rich clinical data are laborious to collect but are critical to understand and adequately resource SDEC services and inform policy; outcomes cannot be directly extrapolated from services for younger patients or pre-selected groups. Our findings will therefore inform development of SDEC focussed on the older acute medical take population and other admission-avoidance models for older patients. Further studies are required on cost-effectiveness and how the multi-faceted dimensions of geriatric care can best be provided taking into account patient views and experiences.

## Supplementary Material

aa_23_0781_File002_afad257
