# Supplementary material for: Care pathways in older patients seen in a multidisciplinary same day emergency care (SDEC) unit
Source: Age Ageing. 2024 Jan 25;53(1):afad257. doi: 10.1093/ageing/afad257 (PMC10811520; doi:10.1093/ageing/afad257)
Supplement: aa_23_0781_File002_afad257 [file aa_23_0781_file002_afad257.docx]

**Care pathways in older patients seen in a multidisciplinary same day emergency care (SDEC) unit**

Tania CN Elias^1^, Chloe Jacklin^2^, Jordan Bowen,^3^ Daniel S Lasserson^3,4,5^, Sarah T Pendlebury^3,6,7^

^1^Departments of Acute Internal Medicine and Geriatric Medicine, Great Western Hospital NHS Foundation Trust, Marlborough Road, Swindon, SN3 6BB

^2^Departments of Care of the Elderly and Stroke Medicine, North Middlesex University Hospital NHS Trust, Sterling Way, London, N18 1QX

^3^Departments of Acute Internal Medicine and Geratology, Oxford University Hospitals NHS Foundation Trust, John Radcliffe Hospital, OX3 9DU

^4^NIHR Applied Research Collaboration (ARC) West Midlands, Warwick Medical School, University of Warwick, Gibbet Hill Campus, Coventry, Warwickshire CV4 3AL

^5^Department of Acute Medicine, City Hospital, Sandwell and West Birmingham Hospitals NHS Trust, B18 7QH

^6^NIHR Oxford Biomedical Research Centre, John Radcliffe Hospital, Oxford University Hospitals NHS Foundation Trust, OX3 9DU

^7^Wolfson Centre for Prevention of Stroke and Dementia, Wolfson Building, Nuffield Department of Clinical Neurosciences, John Radcliffe Hospital, and the University of Oxford

Correspondence to:

Sarah Pendlebury

Wolfson Centre for Prevention of Stroke and Dementia

Wolfson Building

John Radcliffe Hospital

Oxford OX3 9DU

Email: [sarah.pendlebury@ndcn.ox.ac.uk](mailto:sarah.pendlebury@ndcn.ox.ac.uk)

**Appendix Figure**

Number of patients receiving further EMU reviews of any type (face to face, telephone, hospital at home) after initial assessment.

Number of EMU reviews of any type

Number of patients
